# Ronald Fisher and group selection

**DOI:** 10.1007/s40656-025-00691-5

**Published:** 2025-08-27

**Authors:** Robert J. Asher

**Affiliations:** Department of Zoology, University of Cambridge, Downing St., Cambridge, CB2 3EJ UK

**Keywords:** Multi-level selection, Gene's eye view, Inclusive fitness, Human civilization

## Abstract

Ronald Aylmer Fisher (1890–1962) was a pioneer of evolutionary biology who founded modern statistics. He has often been associated with a gene-centric conception of natural selection, but he did not discount the importance of factors operating at other levels. In the later chapters of his 1930 book, *The Genetical Theory of Natural Selection*, Fisher proposed a mechanism to explain how a human civilization of any ethnicity could rise or fall. In contemporary terms, he did this by applying a concept of group selection, also known as “multi-level selection 1”, in which supra-individual collectives impart consistent population structure over time to reproductive entities therein. Fisher believed that socioeconomic factors, operating above the individual level, could bias reproductive patterns and thereby have a causal influence on human social complexity. “Multi-level selection 2” is another kind of group selection which asserts heritable features to units above the level of the individual, and Fisher regarded “sexuality itself” as one such example. Fisher held some inaccurate views about human diversity, but appreciating how his argument foreshadows current multi-level selection theory does not require agreement with his mistakes. The chapters concerning human civilization in *The Genetical Theory* were not a polemic against non-Europeans, but reflected an understanding of multi-level selection and its effects on evolution as a whole.

## Introduction

*The Genetical Theory of Natural Selection* (Fisher, 1930, 1958; Fisher & Bennett, 1999), hereafter referred to as *The Genetical Theory*, was arguably Ronald Fisher’s most important book. In it, Fisher mathematically demonstrated particulate inheritance as a plausible mechanism for adaptation under the Darwin-Wallace theory of evolution. Many previous authors (e.g., Williams, 1966; Dawkins, 1976; Sober, 1984, 2011a; Sober & Wilson, 1998; Leigh, 1999; Borrello, 2010; Harman, 2011; Ågren, 2021) have characterized *The Genetical Theory*, and its author, as the origin of what is now known as the " ‘gene’s eye view’ of evolution, in which everything that evolves is interpreted as a form of ‘genetic selfishness’ " (Wilson & Wilson, 2007, p. 331). *The Genetical Theory* has two major parts (Aylward, 2022): an initial seven chapters on particulate inheritance, genetics, and variation, followed by five chapters on human society. Its initial chapters are generally those which have remained in high regard in virtually all postwar scholarship in biology and statistics. Conversely, Fisher’s chapters on human society are more controversial due to his advocacy of eugenics, i.e., using then-understood principles of Darwin and Mendel to try and address human social needs. Fisher was among many of his generation who, across a broad spectrum of political views, advocated such policies (Freeden, 1979). Once the term “eugenics” became associated with Nazi Germany, such advocacy disappeared, and relatively few authors (e.g., Hamilton, 2001: Chap. 12) considered the word as most pre-war authors (e.g., Fisher, 1935; Huxley, 1936) meant it, and not as a synonym for genocide or murder (see discussion in Cavalli-Sforza & Bodmer, 1971: Chap. 12). For others, “a tradition of discreet silence” (Gould, 2002, p. 512) enveloped Fisher’s later chapters of *The Genetical Theory*. According to Grafen (2003, p. 327),

The final five chapters of Fisher (1930) deal with selection in humans, and Fisher considered these an essential part of the whole. Today’s biologists do not much read those chapters, I guess partly because of fear, essentially of social Darwinism and through an association with genocide, and partly because we are narrower technicians now and tend to suspect grand altruistic applications of our subjects. There is so much still to emerge from chapters [1–7] of Fisher (1930) that it would be surprising if a close study of the remaining, largely neglected, chapters did not yield important ideas.

I believe that a sophisticated application of group selection is among the “important ideas” contained in Fisher’s later chapters. Although the basic principles behind group selection were not new in Fisher’s time (Darwin, 1871, p. 166), and while at least a few writers have noted Fisher’s appreciation of them (e.g., Karlin, 1992, p. 29; Esposito, 2016, 2018), Fisher has been frequently cited as an authority against the group as an important unit of evolutionary selection (Williams, 1966; Sober, 1984, 2011a; Sober & Wilson, 1998; Leigh, 1999; Borrello, 2010; Harman, 2011). A possible explanation for this arises from the fact that Fisher wrote about group selection primarily in the last five chapters of *The Genetical Theory*, exactly those which have been dismissed in the postwar literature (e.g., Sober, 2024, p. 112). In fact, what Fisher wrote about human society foreshadows rather closely mechanisms behind group selection articulated in recent decades (e.g., Rice, 2004; Okasha, 2006).

### Group selection

Evolution by natural selection is based on differential reproductive success over time. “Fitness” is commonly defined in this sense; the more offspring you contribute, the fitter you are. Yet there are many examples of traits and behaviors that seem to betray individual fitness and imply something acting on the group as a whole. Social insects forego their own reproduction to help raise the offspring of their queen. Individuals in a collective may engage in alarm calls that, while alerting others to an approaching predator, put themselves in danger. Group behaviors such as a murmuration of starlings, or coordinated movements in a school of fish, suggest a level of cognitive interaction that would, at first glance, only seem possible with some capacity for group-level adaptation.

Evolutionary explanations of group-level phenomena prior to the mid-1960s (e.g., Emerson, 1960; Wynne-Edwards, 1962) often made appeals to the “good of the group”, for example in reference to species lowering its reproductive potential in anticipation of future resource scarcity. Fisher has never been associated with this kind of “naive” group selection (see Wilson, 1983), and his ideas about group-level phenomena formed the basis by which subsequent authors (e.g., Hamilton, 1964; Maynard Smith, 1964, 1976; Williams, 1966; Dawkins, 1976) developed more gene-centric theories. Especially after Williams’ (1966) influential book *Adaptation and Natural Selection*, the concept of group selection was unpopular for much of the late 20th century (Borrello, 2010). Recent decades have witnessed a softening of this resistance and an increasing acknowledgment of its theoretical utility (Hamilton, 1975; Wade, 1985; Sober & Wilson, 1998; Rice, 2004; Okasha, 2006; Wilson & Wilson, 2007), although debate remains about several aspects of how group selection should be interpreted (e.g., Gardner & Grafen, 2009; Sober, 2011b; Okasha, 2011, 2024; Okasha & Paternotte, 2012; Goodnight, 2015; Gardner, 2015a, b).

While groups themselves are not typically regarded as reproductive entities, they can significantly impact how individuals reproduce. The influence of group dynamics on individual fitness can be framed in terms of frequency dependence, without necessarily invoking group selection. However, when groups exhibit varying degrees of coherence and longevity, such that some proliferate more than others over time, higher fitness of such groups, with particular combinations of individuals, can ultimately have a major influence on phenotype and behavior. Evolution therefore cannot be properly understood without an appreciation of the group level conditions that influence individual reproduction. Broadly speaking, the individual and group are just two of many potential levels at which selection can operate. Such levels can be above (e.g., a hive) or below (e.g., a gamete) a sexually reproducing individual (Lloyd, 2001; Rice, 2004; Okasha, 2006). Multi-level selection is the larger concept within which group selection fits, where a group is a level of biological organization containing reproductive individuals. Sober and Wilson (1998) used an example from poultry farming to illustrate evolution at one such level:

Selecting for increased egg production at the individual level can actually reduce the productivity of individuals.… Hens may have been most productive because they are the best egg-laying machines [or] because they are the nastiest hens in the flock and were able to suppress the production of other hens.…To select for chickens as *social* egg-laying machines, it is necessary to select at the level of whole chicken societies.… Groups of hens… were scored for egg production [by Muir, 1996] and hens from the most productive groups were used as breeders for the next generation of groups. The response to group selection was… dramatic.… Annual egg production increased 160% in only 6 generations. (Sober & Wilson, 1998, p. 141, italics original)

### Fisher’s “human” chapters and levels of selection

Fisher’s ideas concerning human fertility and social success in chapters VIII-XII of *The Genetical Theory* entailed dynamics similar to Sober and Wilson’s (1998) example of poultry farming. In order to explain the rise and fall of any human civilization, Fisher considered how social success correlated with high fertility within human cultures. Their co-occurrence, he thought, explained the rise of civilization:

There have certainly existed societies, though not properly speaking civilized societies, in which the institution of social class was highly developed, in which the power and prestige of the individual rested largely upon his pedigree and kinship, in which these class distinctions were doubtless correlated with personal wealth, and in which, nevertheless, the social advantage lay with the larger families. Since in such societies we should infer from the principle of the inheritance of fertility, and the absence of any countervailing causes, that the fertility of the socially superior should be the higher, and consequently that the powerful evolutionary force, which such difference of fertility has been shown to exert, will be directed towards the increase of the qualities favourable to success in these societies, and to the qualities admired in them, their importance for the study of our theory, and for the evolutionary history of mankind, is very great. (Fisher, 1930, pp. 242–243)

When the two did not correspond, he thought social collapse would result:

wherever, then, the socially lower occupations are the more fertile, we must face the paradox that the biologically successful members of our society are to be found principally among its social failures, and equally that classes of persons who are prosperous and socially successful are, on the whole, the biological failures…. The struggle for existence is, within such societies, the inverse of the struggle for property and power.… In societies so constituted, we have evidence of the absolute failure of the economic system to reconcile the practice of individual reproduction with the permanent existence of a population fit, by their mutual services, for existence in society. (Fisher, 1930, pp. 221–222)

Although Fisher likely overstated the heritable basis of “social failure” and “success”, he still recognized that conditions within a group can influence individual fitness (as more recently explored by, for example, Heyer et al., 2005 and Guez et al., 2023), and he recognized that the nature of such conditions may vary across competing human societies, or units within a single society. Fisher’s view of socioeconomic class as an organizational principle by which individuals form groups is an example of what Okasha (2006, p. 58, following Damuth & Heisler, 1988) referred to as “multi-level selection 1” (MLS1), which focuses on the units of inheritance (i.e., genes, hereafter referred to as “particles” following Okasha, 2006) within the group. This contrasts with “multi-level selection 2” (MLS2), which focuses on the group itself, for example species in the fossil record, which may themselves have heritable properties, such as geographic range (Jablonski, 1987). Both Sober & Wilson’s (1998) example of poultry and Fisher’s (1930) application of socioeconomic class to human fertility concern MLS1. In both cases,

the role of the collectives in MLS1 is to generate population structure for the particles, which affects their fitnesses. For MLS1 to produce sustained evolutionary consequences, collectives must ‘reappear’ regularly down the generations, but there is no reason why the collectives themselves must stand in parent–offspring relations. (Okasha, 2006, p. 58)

In poultry, collectives are the breeding groups imposed by human managers (Muir, 1996). In humans, they may include language groups or socioeconomic classes. In either case, the collective is not itself a reproductive entity, but is sufficiently coherent and long-lived so as to consistently affect individual fitness over many generations, and thereby have the potential to “produce sustained evolutionary consequences”.

Some authors have expressed disappointment with this concept of group selection because it still boils down to the differential reproduction of individuals, with added terminology (or more pejoratively, jargon) for what might be more simply known as frequency-dependent selection. For example, “the argument is not about what the world is like, or even about how we should model it… but about what words we should use to explain our model” (Maynard-Smith 1998, p. 640). Such a view holds that, even with “sustained evolutionary consequences”, the frequency dependence implied by MLS1 should still be called “individual selection”. MLS2, on the other hand, is a stronger claim about a parent-offspring relationship between collectives themselves (Okasha, 2006, p. 58).

### Fisher’s mixed signals about groups

There are a few passages in *The Genetical Theory* in which Fisher appeared to simultaneously endorse yet diminish the importance of group selection. I believe the distinction between MLS1 and MLS2 helps to understand these apparent contradictions. For example,

[If] selection would tend to increase [the most eminent families’] innate fertility above that of the less distinguished groups, it is not surprising that we should be led to conclude that society was derived generation after generation, predominantly from its more successful members. The most important consequence of this conclusion is that human evolution, at least in certain very ancient states of society, has proceeded by an agency much more powerful than the direct selection of individuals, namely the social promotion of fertility into the superior social strata. (Fisher, 1930, p. 245)

As Fisher saw them, “social strata” provided structures that can consistently influence how particles (sensu Okasha, 2006) are transmitted over many generations, therefore comprising an MLS1 mechanism by which any human society might evolve. In Fisher’s words, this mechanism is “much more powerful than the direct selection of individuals”. His “social strata” are not themselves distinct societies but units therein, with distinct capacities for proliferation over time.

Immediately following this passage, Fisher then seemed to express skepticism about group selection:

[I]t is important that the qualities recognized by man as socially valuable, should have been the objective of such a selective agency, for it has hitherto only been possible to ascribe their evolutionary development to the selection of whole organized groups, comparable to the hives of the social insects. The selection of whole groups is, however, a much slower process than the selection of individuals, and in view of the length of the generation in man the evolution of his higher mental faculties, and especially of the self-sacrificing element in his moral nature, would seem to require the action of group selection over an immense period. (Fisher, 1930, p. 245)

His phrase “selection of whole groups” implies an MLS2 mechanism, or collectives that give rise to other collectives in a parent-offspring relationship “as evolving units in their own right, not just as part of the particles’ environment” (Okasha, 2006, p. 56). Under this interpretation, Fisher’s view is that “the social promotion of fertility”, an MLS1 mechanism, is “much more powerful than the direct selection of individuals” and preferable to “the selection of whole groups”, an MLS2 mechanism, which would require “an immense period” to have an appreciable evolutionary effect.

Notably, Fisher did not discount MLS2. In the 1958 edition of *The Genetical Theory* (the 1999 variorum edition of which is cited here) Fisher added a section at the end of chapter two, entitled “The benefit of the species”, which started with the observation that

the principle of Natural Selection, in the form in which it has been stated in [*The Genetical Theory* Chapter two], refers only to the variation among individuals (or co-operative communities)… only in so far as variations… are of advantage to the individual.… It affords no corresponding explanation for any properties… which, without being individually advantageous, are supposed to be of service to the species to which they belong.… There would, however, be some warrant on historical grounds for saying that the term Natural Selection should include, not only the selective survival of individuals of the same species, but of mutually competing species of the same genus or family. (Fisher & Bennett, 1999, pp. 279–280)

Here, Fisher is endorsing the idea that “selective survival… of mutually competing species of the same genus or family” should be considered a part of Natural Selection. This passage echoes a 1912 essay in which Fisher discussed social insects as an example of “inter-communal competition”. The hive forms a collective with a given lifespan and fitness, itself subject to competition and differential survival relative to other such collectives:

This process of co-ordination, of integration of units into societies has been carried a step further among the social insects… Queens and drones are produced which by their union cause an immense increase in the number of insects and finally [lead] to the production of a fresh hive. In these respects an ant hive is very similar to, for instance, a fresh water polyp. The subjection of the ants on the one hand, and the cells on the other, to the needs of the whole, has in both cases been established by inter-communal competition. The complicated and highly perfected instincts of the workers have been produced by the natural selection of those hives in which these instincts were well developed.… Among social insects the instinct of self-sacrifice may be completely developed, since the only chance of reproduction lies in the survival of the hive. (Fisher 1912, from Bennett, 1983, p. 60)

Fisher’s reference here to “inter-communal competition” implies an MLS2 mechanism, and parallels more recent definitions of MLS2 “fitness” in terms of the number of collective offspring, not individual offspring influenced by group-level conditions (Okasha, 2006, p. 58). Here, MLS2 obtains when specialization channels reproduction towards certain parts of the whole, e.g., social insect queens or cellular gametes. Fisher then made a direct comparison between social insect and human society:

Human societies are not so far developed as those of insects, and are very far from the complete cell-socialism of the animal body; still it is obvious that the best organized will survive, those in which every class is well cared for, and correspondingly every class performs its functions regularly and without interruption; the nations in which moreover there exist highly skilled and efficient specialized members will reach a higher degree of organization than those in which the members are unskilled although efficient in a general way.(Fisher 1912, from Bennett, 1983, pp. 60–61)

Yet Fisher’s conclusion to his 1958 “benefit of the species” section seemed to downplay group selection:

The relative unimportance of [selection on genera or families] as an evolutionary factor would seem to follow decisively from the small *number* of closely related species which in fact do come into competition, as compared to the number of individuals in the same species; and from the vastly greater *duration* of the species compared to the individual. Any characters ascribed to interspecific selection should of course characterize, not species, but whole genera or families, and it may be doubted if it would be possible to point to any such character, with the possible exception… of sexuality itself, which could be interpreted as evolved for the specific rather than for the individual advantage. (Fisher & Bennett, 1999, p. 280, emphasis original)

As with the passage cited earlier (Fisher, 1930, p. 245), Fisher seemed to contradict what he had previously written on the importance of groups. Again, I believe the apparent contradiction results from a distinction Fisher made between what we now know as MLS1 and MLS2, long before these terms were defined by Damuth and Heisler (1988). Fisher recognized that both can occur, and in the case of MLS2, result in “sexuality itself”, possibly the only character that “evolved for the specific rather than for the individual advantage” (Fisher & Bennett, 1999, p. 280). Here, Fisher’s views presage those of Hamilton (1980, 1982; see also Hamilton, 2001: Chaps. 1, 2 and 11) and Maynard-Smith and Dawkins (1997) on sexual reproduction. Maynard-Smith (in Maynard-Smith & Dawkins 1997) noted that, with few exceptions, asexual species generally appear nested within larger clades dominated by sexually reproducing species, as opposed to sexual species occurring within larger clades of asexuals. Asexuals forego the dual-cost of sex, in which only half of the population reproduces, and each does so by contributing only half of her genes. While such parthenogenic reproduction circumvents this cost, populations then become more susceptible to parasites, predators, and the accumulation of deleterious mutations (Hamilton, 1982; Crow, 1994).

The sporadic presence of asexuals within larger clades of sexual groups suggests that the increased evolutionary rate conferred by sexual reproduction (by definition a supra-individual trait) is a fundamental adaptation across multicellular life. Parthenogenic groups do, on occasion, outcompete sexual groups (and indeed some species can switch between the two modes), but only as long as predators and parasites have not adapted to their weaknesses, and only as long as they have not been swamped by the natural accumulation of deleterious mutations inherent in any evolving population. Conditions exist under which both asexuality and sexuality can succeed, but given the sporadic phylogenetic distribution of the former within sexual clades, and not the converse, sexuality seems to be the more fundamental, group-level adaptation towards accelerating the responsiveness of evolution.

The difference between MLS1 and MLS2 can be further illustrated by contrasting an evolutionary understanding of the origin of sex with maintenance of a 50% sex ratio. As determined by Fisher (1930, pp. 142–143; see also Williams, 1966: Fig. 3), the latter is a frequency-dependent, individual-level adaptation. Whenever sex ratios stray beyond parity, the offspring of individual mothers who tend to birth the rare sex will be at a selective advantage until that sex is no longer rare. While the selective advantage of being a member of the rare sex is at the individual level, a sex-ratio itself is a fundamentally group-level feature. The proportion of males to females is an aspect of the collective that “generate[s] population structure for the particles, which affects their fitnesses” (Okasha, 2006, p. 58) and therefore fits an MLS1 definition. Maynard-Smith’s (1998, p. 640) objection cited above remains, i.e., that calling this “MLS1” is to a considerable extent semantic. However, MLS1 and the individual fitness consequences of frequency-dependence focus on distinct evolutionary problems, each worthy of explanation. Following Okasha (2006, p. 59), “although the explanatory target of an MLS1 model is the change in frequency of a particle character, not a collective character, this does not mean that MLS1 can tell us nothing about the collectives themselves.”

Hence, if Fisher were writing today, he might have considered the origins and maintenance of sexual reproduction as examples of MLS2, and the tendency towards an even sex-ratio as MLS1. Insofar as sex entails the inability of certain individuals (males) to directly generate offspring, it comprises an intermediate condition towards more extreme reproductive specializations, such as haploid gametes among many diploid autosomes, or one reproductive queen among many workers. It is tempting to further speculate if, in addition to the origin of sex, Fisher might have regarded worker sterility and apoptosis (see Okasha, 2024) as MLS2 traits, in line with his “complete cell socialism” from the passage quoted above (Fisher, 1912, from Bennett, 1983, pp. 60–61).

### Groups and the Fundamental Theorem

To appreciate how Fisher has become closely associated with the “gene’s eye view”, it is helpful to consider his Fundamental Theorem of Natural Selection (Ewens, 1989; Edwards, 2014). In *The Genetical Theory*, Fisher (1930, p. 35) expressed his Theorem in plain English: “The rate of increase in fitness of any organism at any time is equal to its genetic variance in fitness at that time”. Consequently, selection applied to a population with more additive genetic variance will increase fitness more quickly than selection applied to a population with less additive genetic variance (Price, 1972a). It doesn’t matter if reproductive patterns are biased at one level or another. All other things equal, higher variability among particles at one time will result in a faster rate at which, under selection, descendants of those particles will change over time. Fisher’s focus on small units of inheritance and rate of change was not a denial of multi-level selection or other phenomena (Charlesworth, 2000; Frank, 2012), but reflected his apparent desire to frame his Fundamental Theorem as an overarching principle, analogous to laws in physics (thermodynamics) or chemistry (gases). Given the object of explanation (the relationship of variation to rate of change), high-level associations of particles were irrelevant. This does make Fisher’s Fundamental Theorem “gene centered”, but

the expression ‘gene-centred’ was not used by Fisher… He was not simply attaching fitnesses to genes and ignoring all questions of interaction and other non-linear components.… In evaluating a population by its gene frequencies Fisher was not ignoring its other features but concentrating on the one defining feature capable of rigorous analysis.… The point of view is indeed that of the ‘gene’s eye’, but the scenery is forever changing. (Edwards, 2014, p. 136)

Considering Fisher’s interest in describing the rate of change, the author of this quote (Edwards, 2014) then cites Gayon (1998, p. 326): “There is no place for ‘levels’ of selection in such a representation of evolution. There is only one kind of selection, the selection of genes.”

Given this characterization of his Fundamental Theorem, Fisher has often been associated with the revival against group selection, particularly in the years after Williams (1966). For example, Harman (2011, p. 170) wrote that “Williams found what he needed to ‘kill group selection’ buried in a paragraph in Fisher (1930)”. Harman was referring to Fisher’s (1930, pp. 158–159) discussion of mimicry in butterflies, in which Fisher observed that advantageous traits of individual butterflies can be transmitted across generations not only via their own offspring, but also via offspring of their genetically and phenotypically similar siblings. This presages Hamilton’s (1964) kin-based concept of inclusive fitness (see “Altruism”, below).

A Fisherian view of evolution has often been contrasted with group-level concepts inherent in Sewall Wright’s metaphor of the adaptive landscape (e.g., Gayon, 1998, pp. 325, 354), which entailed the differential survival of groups small enough to be dominated by genetic drift. Wright’s landscape is meant to represent a three-dimensional area in which fitness changes along the Y-axis. Assuming a sufficient effective population size, selection will pull a population upwards towards higher fitness on any local peak, however suboptimal that peak may be relative to others on the overall landscape. Wright believed that, in order to cross fitness valleys between peaks, a species would have to be divided into small groups, i.e., populations with low effective population size, in which drift plays a stronger role than selection.

Fisher’s dislike of the adaptive landscape metaphor is well known (Provine, 1986; Ewens, 1989; Crow, 1990; Frank & Slatkin, 1992; Frank, 2012), and his antipathy towards such ideas from Wright probably amplified his reputation as a champion of the “gene’s eye view”. His feud with Wright may also have influenced Fisher to word his “benefit of the species” section in the 1958 edition of *The Genetical Theory* so as to further downplay group-level effects. In fact, while many have perceived Fisher as epitomizing the gene’s eye view, and while his Fundamental Theorem is logically based in it (Okasha, 2008), Fisher had no difficulty incorporating the effects of either group-dynamics on individual fitness, or differential survival of some groups over others, into his overall understanding of the evolutionary process (Edwards, 2011, 2014; Esposito, 2016, 2018; Grodwohl, 2018). Fisher’s identification of links between fertility and social dynamics among humans, however (in)accurate his views about human diversity or the heritability of social traits may have been, demonstrates his recognition of the kind of group selection known today as MLS1.

### Altruism

The evolution of cooperative behavior that comes at an individual cost, or altruism, is usually invoked in discussions of group selection. When cooperative behavior is sufficiently widespread in a group, that group may have higher fitness compared to those in which cooperative behavior is absent or rare, despite the fact that selection favors selfish individuals within each group. One famous resolution of this paradox comes in the form of inclusive fitness (Hamilton, 1964), in which altruists direct their cooperative behavior towards close genetic relatives, such as siblings of a distasteful butterfly larva. The naive predator who attacks it will thereby learn to avoid any sensory cues associated with the entire brood of “gregarious larvae” (Fisher, 1930, p. 159). The attacked individual thus exhibits “altruism” towards its genetically similar siblings.

In this light, what at first seems to be “altruism” turns out to be genetic nepotism. An individual sacrifices itself in favor of a close relative only because of the consequent increase of particles in which both have an interest. Terms like “selfish” or “altruistic” become anthropomorphic reflections of the perspective we take. Particles are “selfish” insofar as they manipulate “altruistic” behavior at higher levels of organization in which those particles are distributed; individuals are not really “altruistic” due to their common investment in a given set of particles.

Yet such emotive terms distract from the more basic point: bias in reproductive pattern across individuals is an important influence on how heritable particles are distributed in future generations. Moreover, kin-recognition is just one of many ways in which inter-individual reproductive patterns can be biased. From this point of view, kin-based inclusive fitness, usually attributed to Hamilton (1964), is a special case of multi-level selection (Price, 1972b; Rice, 2004; Okasha, 2006). As Hamilton himself noted,

kinship should be considered just one way of getting positive regression of genotype in the recipient.… [It] is this positive regression that is vitally necessary for altruism. Thus the inclusive-fitness concept is more general than ‘kin-selection’.… [It] makes no difference if altruists settle with altruists because they are related… or because they recognize fellow altruists as such, or settle together because of some pleiotropic effect of the gene on habitat preference. (Hamilton, 1975, reprinted in Hamilton, 1996, p. 337)

It is therefore possible to understand the evolution of altruism via multi-level selection (see also Hamilton, 1996, p. 324), whatever the genetic relatedness among altruists may be.

In Chap. 11 of *The Genetical Theory*, Fisher applied what we would now call inclusive fitness to explain the evolution of “heroism”, or a suite of altruistic behaviors among humans:

An examination of the action of selection in barbarous societies in the tribal condition, reveals the possibility of the selection of the heroic qualities beyond the limits set by prudence, by a method analogous to that used in Chapter VII to explain the evolution of distasteful qualities in insect larvae. The mere fact that the prosperity of the group is at stake makes the sacrifice of individual lives occasionally advantageous, though this, I believe, is a minor consideration compared with the enormous advantage conferred by the prestige of the hero upon all his kinsmen. (Fisher, 1930, p. 246)

While Fisher’s early 20th century vocabulary may appear awkward to contemporary readers, he is nonetheless applying inclusive fitness (“a method analogous to… explain the evolution of distasteful qualities in insect larvae”) to understand altruism (“selection of the heroic qualities beyond the limits set by prudence”). Furthermore, although the term “kinsmen” implies a genetic basis for social association, it is not the only one, as noted by Hamilton (1975, 1996). Finally, there are elements of both MLS1 and MLS2 in this passage, and Fisher emphasizes the former. “Prosperity of the group”, implying MLS2, is duly noted, but as “a minor consideration compared with the enormous advantage conferred by the prestige of the hero”. This “prestige” is a social factor that can align reproductive with socioeconomic success in some human social units, but not others, and comprises the MLS1 basis of group selection, as noted above. According to Fisher, therefore, supra-individual collectives within a society (or between societies) can “generate population structure for the particles, which affects their fitnesses” (Okasha, 2006, p. 58) by aligning (in the “tribal condition”) or separating (in established civilizations) social with reproductive success.

### Class, not “race”

Early reviewers of *The Genetical Theory* included Fisher’s North American intellectual competitor Sewall Wright and the polymath geneticist (and unapologetically communist) JBS Haldane. Both had critiques, but still recognized Fisher’s seminal accomplishment. Haldane (1931, pp. 116–117) in particular did not shy away from stating what he liked about Fisher’s chapters on human civilization. With the postwar association of eugenics with Nazism, this kind of positive assessment disappeared (e.g., Gould, 2002, p. 512; Grafen, 2003, p. 327). An author who believes that Fisher advocated white supremacy or Nazism may very well regard his chapters on human civilization as “an embarrassment” (Sober, 2024, p. 112), and not be inclined to consider how those chapters relate to multi-level selection.

Fisher did not advocate either white supremacy or Nazism in *The Genetical Theory*, but claims or implications to the contrary nonetheless exist in the recent literature. For example, without questioning its veracity, the emeritus Regius Professor of History at the University of Cambridge, Evans (2020), repeated text from an online petition (Donlon-Mansbridge, 2020), in turn based on a blog post (Joselson, 2016), to the effect that in *The Genetical Theory*, Fisher

wrote that civilisations fail because people of ‘low genetic value’ [read black and brown people] have more children than people with ‘high genetic value’” (read white Europeans) and said that this was already happening in Great Britain. (Evans, 2020, parentheticals original)

Similarly, the BBC (2020) reported that Fisher “was known for arguing people were divided into genetically inferior and superior groups along racial lines”. Such an allegation fits into the narrative that Fisher sympathized with Nazi atrocities, as implied by Evans (2020) and Rutherford (2021; but see Bodmer et al., 2021; Edwards, 2024a, b).

In fact, nowhere in *The Genetical Theory* did Fisher attribute genetic superiority to any particular ethnic group. By “genetic value”, Fisher (1930) was referring to genetic variance and the potential for some alleles to exhibit dominance (p. 33), or methods to uncover a genetic basis for continuous variation (p. 112). Moreover, Fisher (1930, p. 241) used the phrase “inferior race” in *The Genetical Theory* hypothetically, not in reference to ethnic features such as melanic skin. Instead, he was referring to European populations such as those who might have (but did not) intermix with Roman settlements in France and Britain (Fisher, 1930, p. 241). Furthermore, Fisher (1930, p. 239) argued *against* the views of an actual white-supremacist, J.A. Gobineau, who claimed that civilizations formed only under the beneficent influence of white Europeans and collapsed due to subsequent “racial mixture”. Disputing Gobineau’s white-supremacist views, Fisher wrote that

it remains to be explained why great civilizations should be assumed always to rest upon so precarious a foundation. If the peoples of the world’s great centres of ancient population are inherently incapable of producing of themselves a great and vigorous civilization, an explanation of so remarkable a fact is certainly required. (Fisher, 1930, p. 239)

Fisher did not accept Gobineau’s idea that the great human civilizations were dependent on the qualities of a non-indigenous population (see also Esposito, 2018). Gobineau’s claim rested “on so precarious a foundation” and, if true, “an explanation… is certainly required”, but Gobineau provided none.

Like many authors of the past, Fisher took seriously certain inaccurate ideas, not least the possibility that modern human ethnicities are more biological than social categories. His personal correspondence suggests he did believe that human ethnicities varied in terms of “their innate capacity for intellectual and emotional development” (Fisher, 1951). However, his views must be considered in the context of the past century, when overtly bigoted, public statements by academics about human ethnicity were not rare (e.g., Osborn, 1926; Weinert in UNESCO, 1952, p. 35; George, 1962), and when an understanding of the origins and recent dispersal of *Homo sapiens* from Africa was generally lacking. Fisher’s published statements on human ethnicity were markedly different from those of, say, Osborn (1926) and George (1962), not to mention his track record as an academic mentor (Bodmer et al., 2021). For example, in regards to interracial marriage, Fisher wrote

If the races and their descendants intermarry freely, the factors which are inherited independently will be recombined at random… Their general character will therefore be intermediate, but their variability will be greater than that of the original races. Moreover, new combinations of virtue and ability, and of their opposites, will appear in the mixed race… There are thus in the mixed race great possibilities for the action of selection. If selection is beneficent, and the better types leave the greater number of descendants, the ultimate effect of mixture will be the production of a race, not inferior to either of those from which it sprang, but rather superior to both, in so far as the advantages of both can be combined. Unfavourable selection, on the other hand, will be more rapidly disastrous to a mixed race than to its progenitors. (Fisher, 1930, p. 238)

Such a viewpoint is obviously not a modern one, but Fisher’s willingness to consider intermarriage that results in “a race, not inferior to either of those from which it sprang, but rather superior to both” shows that he was not a white supremacist.

Despite its antiquated style, *The Genetical Theory* was not a polemic against non-Europeans. The whole point of his final chapters was to provide what Gobineau and others like him did not: a theory of the rise and fall of civilization, no less applicable to the endemic populations of Africa and Asia than to those of Greece, Rome, or indeed his own Britain. Assuming, as Fisher did, that “economically successful” correlated with what he considered to be traits with at least some heritable basis such as intelligence, bravery, or work ethic, the pattern over time was clear: whether it takes place in Egypt or England, societies are doomed unless they re-establish correlation of economic with reproductive success. Fisher did not use ethnic criteria as determinants of “genetic value”; on the contrary, “the experiment of becoming civilized has in fact been performed repeatedly by peoples of very different races” (Fisher, 1930, p. 176). His theory of the rise and fall of human civilization was based on what he perceived to be differential fertility among groups varying in socioeconomic class, not skin color.

## Conclusions

Fisher’s last five chapters in *The Genetical Theory* proposed a group-level, MLS1 mechanism by which any human society might rise or fall, whether it was in Egypt, China, India, Rome, or anywhere else. This recognition is not dependent on the accuracy of Fisher’s assumptions about human ethnicity or the heritability of social traits. Rather, it is the structure of his argument that makes it relevant to contemporary multi-level selection theory. Fisher evidently believed that socially valuable traits have a substantial heritable basis. As a result, when collectives within a society in possession of such traits have disproportionately more (or fewer) children, then that society has an ever-increasing (or decreasing) chance at success as Fisher knew it; i.e., it is apt to become “civilized” (or fall). Setting aside the point, never discussed by Fisher, that “civilized” is not necessarily better in terms of individual quality of life (see Graeber & Wengrow, 2021), Fisher did not reserve the phrase “superior social strata” for any particular human ethnicity or skin color.

It is not necessary to overlook Fisher’s various mistakes (e.g., Stolley, 1991) to recognize that *The Genetical Theory* has a substantial basis in contemporary multilevel selection theory. Ronald Fisher was a scientific visionary who pioneered much of contemporary statistics and evolutionary biology. Further insights from his scholarship continue to be discovered, an example of which is his appreciation of group selection in the later chapters of *The Genetical Theory*.

## Data Availability

Not applicable.
